# Mechanisms of hypothermia-induced cell protection in the brain

**DOI:** 10.1186/s40348-014-0007-x

**Published:** 2014-12-01

**Authors:** Katharina Rose Luise Schmitt, Giang Tong, Felix Berger

**Affiliations:** Department of Congenital Heart Disease/Pediatric Cardiology, German Heart Institute Berlin, Augustenburger Platz 1, 13353 Berlin, Germany; Department of Pediatric Cardiology, Charité Universitätsmedizin Berlin, Campus Virchow-Klinikum, Augustenburger Platz 1, 13353 Berlin, Germany

**Keywords:** Hypothermia, Neuroprotection, RBM3, Anoxic injury, Inflammation

## Abstract

Therapeutic hypothermia is an effective cytoprotectant and promising intervention shown to improve outcome in patients following cardiac arrest and neonatal hypoxia-ischemia. However, despite our clinical and experimental experiences, the protective molecular mechanisms of therapeutic hypothermia remain to be elucidated. Therefore, in this brief overview we discuss both the clinical evidence and molecular mechanisms of therapeutic hypothermia in order to provide further insights into this promising intervention.

## Introduction

Clinicians have investigated the application of therapeutic hypothermia on the human body for centuries. Increasing clinical evidence from meta-analysis of large randomized controlled trials and experimental data advocates the induction of therapeutic hypothermia as a tool to achieve neuroprotection [1]. Clinical indications for therapeutic hypothermia as a protection strategy include: myocardial infarction [2,3], cardiopulmonary bypass in adults [4], pediatric open heart surgery [5,6], stroke [7], neonatal hypoxia-ischemia [8-10], and traumatic brain injury [11,12].

To date, the strongest evidence for its efficacy exists for various clinical situations following cardiac arrest and neonatal hypoxia-ischemia. However, the effective application of hypothermia requires a thorough understanding of the injury mechanisms as well as its protective mechanisms. Therefore, we discuss in this brief overview both the clinical factors and molecular mechanisms of therapeutic hypothermia in order to provide further insights into this promising intervention.

## Review

### Therapeutic hypothermia: clinical evidence

Early experiences with therapeutic hypothermia in the 1940s thru 1960s falsely assumed that the protective effects were only due to the temperature-dependent reduction in metabolism, which leads to lower oxygen and glucose demands [13]. Therefore, patients were routinely subjected to deep hypothermia (<30°C) with varying durations ranging from 2 to 10 days [14]. Animal experiments in the 1980s led to the breakthrough discovery that using mild to moderate hypothermia (31°C to 35°C) resulted in improved neurological outcome with fewer and less severe side effects [15]. More importantly, these findings led to the realization that hypothermia-induced neuroprotection is not only limited to decreased oxygen and glucose demands, but the mechanisms involved are indeed much more intricate.

Three large multicenter randomized studies of newborn infants with hypoxic ischemic encephalopathy suggest a beneficial effect in this patient population. Gluckman et al. demonstrated an improved outcome that persisted at 18 months of life in term infants suffering from moderate neonatal encephalopathy who were subjected to head cooling (CoolCap, Natus, San Carlos, CA, USA) for 72 h [10]. A second trial demonstrated whole-body cooling to 33.5°C for 72 h reduced the risk of mortality or moderate to severe disability in infants with moderate or severe encephalopathy surveyed at 18 to 22 months of age [16]. A third published Total Body Hypothermia for Neonatal Encephalopathy (TOBY) trial also showed benefits from similar whole-body cooling in newborns with perinatal asphyxia [17]. The study showed that hypothermia did not significantly reduce the rate of mortality or severe disability but resulted in improved neurologic outcomes in infants assessed at 18 months of age. However, the criteria for optimal candidates for therapeutic hypothermia have yet to be defined, and long-term follow-up (beyond 18 months of age) to assess the persistent and lifelong benefits are needed.

Current investigations also include the Infant Cooling Evaluation (ICE) trial to investigate the effect of moderate whole-body hypothermia to 33.5°C for 72 h in newborns with HIE [18] and the Therapeutic Hypothermia after Pediatric Cardiac Arrest (THAPCA) trials, a 30-site randomized clinical trial investigating the effectiveness of therapeutic hypothermia versus therapeutic normothermia after in-hospital (THAPCA-IH) or out-of-hospital (THAPCA-OH) cardiac arrest in children [19,20].

#### Clinical issues

Clinical issues regarding optimal target temperature, rate of cooling, duration of cooling, rate of rewarming, as well as optimal treatment window need further investigation. With the introduction of therapeutic hypothermia, it is important to differentiate between the ‘induction phase’ , when the temperature drops; the ‘maintenance phase’ , when the target temperature is achieved and maintained at the desired level; and the ‘rewarming phase’ , when the patient is slowly rewarmed back to normothermia.

Hypothermia induces stress responses, such as shivering, that result in increased oxygen consumption and metabolic rate in non-sedated patients. Animal experiments suggest that the neuroprotective effects of hypothermia are negated if cooling is used on non-sedated animals [21,22]. Other side effects include increased risk of infection, cold diuresis and hypovolemia, electrolyte disorders, insulin resistance, impaired drug clearance, and mild coagulopathy [15].

### Therapeutic hypothermia: molecular mechanisms

Therapeutic hypothermia-induced cellular protection against anoxic brain injury is a global process affecting multiple molecular and cellular mechanisms. Cooling results in a 6% to 10% decrease in cerebral metabolism for every 1°C reduction in body temperature [23]. Therefore, a thorough understanding of the underlying mechanisms of protection induced by therapeutic hypothermia is vital for designing appropriate and effective treatments. Insufficient knowledge of the physiological changes and side effects that occur during mild (34°C to 35.9°C), moderate (32°C to 33.9°C), moderate-deep (30°C to 31.9°C), and deep (<30°C) hypothermia is likely to lead to lower therapeutic efficacy or even failure of treatment [15].

Ischemic brain injury, reperfusion injury, and secondary brain damage are the three main categories of temperature-dependent injury processes that can be effectively mitigated by mild to moderate hypothermia. Due to the broad effects of hypothermia, it is more clinically effective than treatments that focus on blocking just one of these processes.

Variable factors such as type of injury (traumatic versus pure ischemia) and patient physiology (genetic factors, age, gender, etc.) all contribute to the complicated injury processes. Although the window of opportunity to initiate hypothermic treatment as well as the duration of cooling to achieve full efficacy may vary, the success or failure of therapeutic hypothermia treatment is dependent on the following four key factors [24,15]:

1. Rate of cooling - rapid initiation of cooling immediately after injury resulted in better outcomes in animal studies. A concept summarized by the phrase ‘time is brain’.
2. Duration of cooling - dependent of severity of injury and the time allotted to achieve target temperature.
3. Rate of rewarming - slow rewarming is critical to not reactivate initial injury processes.
4. Management and prevention of side effects.

#### Apoptosis and mitochondrial dysfunction

Ischemia/reperfusion injuries can lead to cellular necrosis or apoptosis, also referred to as programmed cell death. The development of apoptosis is dependent on several cellular processes, including mitochondria dysfunction, activation of caspase enzymes, and other cellular energy metabolism disorders. Hypothermia has been observed to affect almost all of these injury processes, thereby preventing the initiation or interrupting the early stages of the apoptotic pathway [25]. The window of opportunity to initiate therapeutic hypothermia to mitigate apoptosis after anoxia is broad, as apoptosis begins relatively late and continues for a long duration. Therefore, mitigating the effects of these injury processes of the apoptotic pathway is a viable treatment for neuroprotection in patients.

Interrupted blood supply to the brain causes an immediate reduction in ATP and phosphocreatine, initiating a switch in intracellular metabolism to anaerobic glycolysis [26]. As a result, intracellular levels of inorganic phosphate, lactate, and H^+^ are dramatically increased, leading to both intracellular and extracellular acidosis and calcium (Ca^2+^) influx into the cells [27]. To further exacerbate the injury process, acidosis and the lack of ATP inhibit cellular mechanisms normally responsible for controlling excessive intracellular Ca^2+^, such as ATP-dependent Na^+^/K^+^ pumps and Na^+^, K^+^, and Ca^+^ channels [28]. The excess influx of Ca^2+^ eventually leads to mitochondria dysfunction and activation of the caspase-9 intrinsic apoptotic pathway. Data from experimental studies have proven that hypothermia has a beneficial impact on the ion pump dysfunction and reduces the influx of calcium into the cells, thereby decreasing neurotoxicity [28,29].

#### Inflammation

Ischemia/reperfusion injury stimulates innate immune responses, which can lead to secondary brain injury. It triggers the release of pro-inflammatory cytokines (i.e. IL-1β, TNFα, and IL-6) [30], the chemokine MCP1 [31] and pro-inflammatory mediators (ROS and NOS) in microglia, and circulating leukocytes [32]. Hypothermia attenuates many aspects of this pro-inflammatory immune response, but it also reduces the expression of some anti-inflammatory cytokines (IL-10 and TGFβ) [33]. Animal studies showed controversial effects of hypothermia on NF-κB, a transcription factor that plays a central role in regulating the inflammatory responses [34-37]. Yenari et al. showed that mild hypothermia decreased NF-κB translocation and activation in rodents [37]. In contrast to Fairchild et al. who showed in cell culture studies after moderate hypothermia a prolonged accumulation of NF-κB in the nucleus. These findings were explained with a delayed phosphorylation of IκBα, the cytosolic inhibitory protein of NF-κB. The Janus kinase (JAK) and signal transducer and activator of transcription pathway (STAT) is a common signaling pathway used by many cytokines. Ischemia leads to a JAK2 and STAT3 activation mediated by IL-6. Inhibiting this pathway resulted in a decreased number of apoptotic cells [34]. Mild hypothermia attenuates STAT3 expression which might be a possible mechanism of hypothermia-induced neuroprotection [38].

#### Blood-brain barrier disruption

Mild to moderate hypothermia has been shown to significantly reduce blood-brain barrier disruptions following ischemia-reperfusion injury by decreasing vascular permeability, resulting in decreased edema formation [39-41]. Specifically, hypothermia suppresses the activation of matrix metalloproteinases (MMPs) responsible for degradation of the extracellular matrix while increasing the expression of endogenous MMP inhibitors, such as metalloproteinase inhibitor 2 (TIMP2) [42]. As a result, the structural proteins and cells that constitute the BBB are preserved and the opening of water channels is prevented.

#### Free radical production

The inflammatory response induced by ischemia/reperfusion injury usually coincides with the production of free oxygen radicals such as superoxide (O_2_^−^), peroxynitrite (NO_2_^−^), hydrogen peroxide (H_2_O_2_), and hydroxyl radicals (OH^−^) [43]. The extent of free radical production following ischemia/reperfusion is so large that protective cellular antioxidant mechanisms are overwhelmed, resulting in peroxidation of lipids, proteins, and nucleic acids [27]. Although hypothermia cannot completely diminish free radical production in injured cells, it can significantly reduce the amount of free radicals enough to allow for the endogenous antioxidant mechanisms to mitigate the oxidative damage. In addition, the suppression of free radical production has been observed to be linearly proportional to the decrease in temperature [28].

#### Rescue gene RBM3

Although hypothermia reduces cellular metabolism and down-regulates global protein synthesis in mammalian cells, a small subset of cold-shock proteins are induced by low temperature. RNA-binding motif protein 3 (RBM3), a member of the glycine-rich RNA-binding protein (GRP) family, is one of the first proteins synthesized in response to hypothermia [44] and hypoxia [45]. Additionally, RBM3 expression is elevated in response to NMDA-type glutamate receptor activation [46]. Mild hypothermia is sufficient to induce the expression of RBM3 [44], while elevated temperature has been shown to suppress RBM3 expression [47]. We observed that RBM3 expression is induced by moderate hypothermia (33.5°C) in murine organotypic hippocampal slice cultures (OHSCs) and hippocampal neurons (HT-22), but not by deep hypothermia (17°C) nor in microglia cells (BV-2) at both cooling temperatures [48].

Although many studies on RBM3 have been focused on their regulation in non-neuronal cells in response to hypothermia and other stress factors [44], there is growing interest in their role as effectors in therapeutic hypothermia-induced neuroprotection. RBM3 is widely expressed during the early stages of brain development, especially in the first to second postnatal weeks, where it is dynamically regulated [49]. Recent studies have concluded that RBM3 has anti-apoptotic functions and can enhance cell proliferation [50]. RBM3 is induced by hypoxia independent of HIF-1 [45], which itself is suppressed by mild hypothermia [51]. Furthermore, RBM3 has been shown to play a major role in promoting translation in neuronal cells, and recently, RBM3 up-regulation in neuronal cells in response to hypothermia has been implicated in hypothermia-induced neuroprotection [52].

#### Drug-induced hypothermia

Drug-induced hypothermia may serve as an alternative to physical hypothermia, which can be technically tedious to apply and may cause serious side effects. Animal studies have shown that hypothermia is induced by synthetic cannabinoid CB1 agonists WIN55212-2 and HU-210. Intramuscular injection of WIN55212-2 induced rapid and prolonged hypothermia in a dose-dependent manner in rats [53], and HU-210 was observed to be protective against ischemic damage by reducing infarct volumes and motor dysfunction [54]. In the same study, HU-210 injected after ischemic onset resulted in deeper and lengthier hypothermia.

## Conclusions

Temperature management continues to play an important role in the treatment of patients suffering from neurological injuries. Therapeutic hypothermia and it's proven ability to attenuate post-ischemic injury represents a quantum leap in the clinical setting [15], but more work is needed, particularly in infants and children suffering from asphyxial cardiac arrest. Additionally, the induced hypothermic effects of cannabinoid CB1 receptor agonists, WIN55212-2 and HU-210, as alternatives to or in combination with therapeutic hypothermia also warrant further investigation. The full beneficial effect of cooling remains to be discovered as we optimize the process by expanding our knowledge of the basic mechanisms, verify the treatment windows, and improve the cooling and monitoring devices in the coming years.
